# Head and neck cancer patients show poor oral health as compared to those with other types of cancer

**DOI:** 10.1186/s12903-023-03356-6

**Published:** 2023-09-06

**Authors:** Hiromi Nishi, Taiji Obayashi, Tsutomu Ueda, Kouji Ohta, Hideo Shigeishi, Syuichi Munenaga, Takashi Kono, Yukio Yoshioka, Masaru Konishi, Ryotaro Taga, Yuya Toigawa, Takako Naruse, Eri Ishida, Eri Tsuboi, Kanae Oda, Kana Dainobu, Tomoko Tokikazu, Kotaro Tanimoto, Naoya Kakimoto, Hiroki Ohge, Hidemi Kurihara, Hiroyuki Kawaguchi

**Affiliations:** 1 Department of General Dentistry, Hiroshima University Hospital, 1-2-3 Kasumi, Minami-Ku, Hiroshima, 734-8553 Japan; 2 Department of Dental Hygiene, Ogaki Women’s College, Gifu, Japan; 3 Department of Otorhinolaryngology, Head and Neck Surgery, Graduate School of Biomedical and Health Sciences, Hiroshima University, Hiroshima, Japan; 4 Department of Public Oral Health, Program of Oral Health Sciences, Hiroshima University, Hiroshima, Japan; 5 Department of Molecular Oral Medicine and Maxillofacial Surgery, Graduate School of Biomedical and Health Sciences, Hiroshima University, Hiroshima, Japan; 6 Department of Oral and Maxillofacial Radiology, Hiroshima University Hospital, Hiroshima, Japan; 7 Department of Program of Dentistry, School of Dentistry, Hiroshima University, Hiroshima, Japan; 8 Department of Oral and Maxillofacial Surgery, Graduate School of Biomedical and Health Sciences, Hiroshima University, Hiroshima, Japan; 9 Department of Orthodontics and Craniofacial Developmental Biology, Graduate School of Biomedical and Health Sciences, Hiroshima University, Hiroshima, Japan; 10 Department of Clinical Practice and Support, Hiroshima University Hospital, Hiroshima, Japan; 11 Department of Oral and Maxillofacial Radiology, Graduate School of Biomedical and Health Sciences, Hiroshima University, Hiroshima, Japan; 12 Department of Infectious Diseases, Hiroshima University Hospital, Hiroshima, Japan; 13 Department of Periodontal Medicine, Division of Applied Life Sciences, Institute of Biomedical and Health Sciences, Hiroshima University, Hiroshima, Japan

**Keywords:** Head and neck cancer, Periodontal inflamed surface area

## Abstract

**Purpose:**

Several studies have found associations between periodontitis and various types of cancer. Since the site of head and neck cancer (HNC) has contiguity or proximity to the oral cavity, it may be particularly influenced by oral inflammation. This study aimed to determine whether HNC patients have poor oral health as compared to those with other types of cancer.

**Methods:**

This study retrospectively examined oral environmental factors including periodontal inflamed surface area (PISA), a new periodontal inflammatory parameter. A total of 1030 cancer patients were divided into the HNC (*n* = 142) and other cancer (*n* = 888) groups. Furthermore, the HNC group was divided into high (*n* = 71) and low (*n* = 71) PISA subgroups, and independent risk factors affecting a high PISA value were investigated.

**Results:**

Multivariate logistic regression analysis showed that number of missing teeth (odds ratio 1.72, 95% CI 1.15–2.56, *P* < 0.01), PISA (odds ratio 1.06, 95% CI 1.03–1.06, *P* < 0.05), and oral bacterial count (odds ratio 1.02, 95% CI 1.01–1.03, *P* < 0.01) were independent factors related to HNC. In addition, multivariate logistic regression analysis indicated that current smoker (odds ratio 7.51, 95% CI 1.63–34.71, *P* < 0.01) and presence of untreated dental caries (odds ratio 3.33, 95% CI 1.23–9.00, *P* < 0.05) were independent risk factors affecting high PISA values in HNC patients.

**Conclusion:**

HNC patients have higher levels of gingival inflammation and poor oral health as compared to patients with other types of cancer, indicating that prompt oral assessment and an effective oral hygiene management plan are needed at the time of HNC diagnosis.

**Supplementary Information:**

The online version contains supplementary material available at 10.1186/s12903-023-03356-6.

## Introduction

Several recent studies have indicated that periodontitis is associated with risk of various types of cancer. A systematic review and meta-analysis of subjects in the United States demonstrated that never smokers and individuals with periodontal disease have a higher risk of developing hematopoietic and lymphatic cancer [1]. In a case–control study, tooth loss and DMFT score results showed that dental caries was associated with risk of gastric adenocarcinoma [2]. Furthermore, periodontal disease was found to be related to increased breast cancer risk in a prospective cohort investigation of postmenopausal women [3], while a significant relationship between periodontitis and incidence of lung cancer was detailed in a systematic review and meta-analysis [4]. On the other hand, head and neck cancer (HNC) is the seventh most common cancer in the world, with the number of cases increasing each year in aging populations [5, 6]. HNC includes all malignancies of the upper aerodigestive tract, the majority of which are of mucosal origin, such as that arising from the paranasal sinuses, nasal cavity, oral cavity, naso-, oro-, and hypo-pharynx, and larynx [7]. Those sites have contiguity or proximity to the oral cavity and may be influenced by oral inflammatory disease [8]. Therefore, periodontitis may be associated with a greater risk for HNC as compared with other types of cancer.

Several other previous cohort and case–control studies, as well as meta-analyses have shown an association of HNC with periodontitis [9–12]. Those evaluated number of lost teeth, periodontal pocket depth, tooth mobility, oral hygiene, annual dentist visits, daily tooth brushing, and radiographic evidence of alveolar bone loss as parameters of periodontitis. However, those factors represent periodontal inflammation, both past and present, are used for determining the prognosis of individual teeth, while they may not be applicable as parameters for current inflammatory activity in periodontal tissues.

Periodontal inflamed surface area (PISA), a new index reported by Nesse et al., is used for quantification of the present inflammatory burden posed by periodontal disease and represents the sum of probing pocket depth in bleeding on probing (BOP)-positive sites for the complete dentition [13]. In recent reports, PISA values for investigating periodontitis in patients with ankylosing spondylitis were positively correlated with periodontal indexes, such as BOP, severity of periodontitis, frequency of *Porphyromonas gingivalis* detection, and presence of that bacterium in serum [14]. Additionally, PISA has been found to be associated with serum C-reactive protein (CRP) level, which indicates systemic inflammation, in septuagenarian patients [15, 16]. Thus, PISA is considered able to show present inflammatory activity in periodontal tissues.

We speculated that HNC patients have increased oral environmental risk factors as compared with other cancer patients. This study was conducted as a retrospective examination to analyze oral environmental factors including PISA in patients with HNC as compared to those with other types of cancer. In addition, independent risk factors with effects on high PISA values in HNC patients were investigated.

## Materials and methods

### Patients and methods

**Fig. 1:**
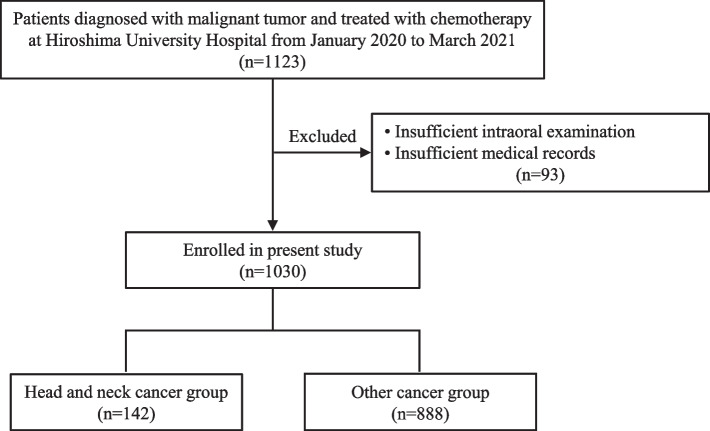
Flow chart showing patient selection for this study

For this registry-based retrospective cohort study, the records of 1123 patients referred to our dental department from a medical department of Hiroshima University Hospital for oral care prior to undergoing chemotherapy for malignant tumors in the period from January 1, 2020 to March 31, 2021 were initially examined. The Strengthening the Reporting of Observational studies in Epidemiology (STROBE) guidelines were used as a checklist [17]. After excluding 93 with poor medical records or an inadequate intraoral examination, 1030 patients (619 males, 411 females) were enrolled, and divided into the HNC and other cancer groups (Fig. 1).

Baseline clinical characteristics for all enrolled patients, including age, gender, smoking status, alcohol consumption, hypertension, diabetes, hyperlipidemia, cerebrovascular disease, cardiovascular disease, history of malignancy, type of cancer, and frequency of dental visits, were collected from electronic medical records. Regarding smoking status, patients with a smoking habit within six months of their first visit were defined as current smokers, those who had quit smoking more than six months prior were defined as former smokers, and those who had never smoked were defined as never-smokers. Hypertension was defined as use of antihypertensive medication or a confirmed resting blood pressure of 140/90 mmHg or higher. Diabetes mellitus was defined as a glycated hemoglobin level of 6.5% or higher, fasting blood glucose level of 126 mg/dl or higher, or use of antidiabetic medication. Hyperlipidemia was defined as total cholesterol level of 220 mg/dl or higher, low-density lipoprotein cholesterol level of 140 mg/dl or higher, high-density lipoprotein cholesterol level of less than 40 mg/dl, triglyceride level of 150 mg/dl or higher, or use of antihyperlipidemic medication. Cerebrovascular disease was defined as history of ischemic stroke, while cardiovascular disease was defined as history of persistent or paroxysmal AF and history of ischemic heart disease, peripheral arterial disease, or congestive heart failure. The attending physician determined the diagnosis of cardiovascular disease.

Approval for this retrospective study (number E-453, registration date April 1, 2019) was obtained from the Epidemiology and Ethics Committee of Hiroshima University. All participants signed an informed consent agreement.

### Oral examination

The enrolled patients were evaluated based on number of missing teeth, presence of untreated dental caries, and periodontal status noted on the day of their first visit for a dental examination, after referral from the medical department and prior to starting chemotherapy. Loss of  ≥ 6 teeth was defined as a tooth loss condition and considered to be associated with risk of HNC [18]. Periodontal inflamed surface area (PISA) was determined using the method reported by Nesse et al*.* and previous studies including our own [13, 14, 19–22]. All measurements were performed using fully erupted teeth at six sites per tooth with a Williams periodontal probe (HuFriedy, USA). Bleeding was recorded as either present or absent within 30 s of probing at all sites of each tooth. The intra-rater reliability of pocket probing was determined prior to the start of the present study. For that determination, probing depth was assessed at six sites (mesiobuccal, mesiolingual, buccal, lingual, distobuccal, distolingual) in three different subjects two separate times by a dentist. The calculated value of the intraclass correlation coefficient suggesting a reproducible assessment of pocket depth was found to be 0.80. PISA is based on the amount of probing pocket depth of sites with bleeding among the total dentition, whereas periodontal epithelial surface area (PESA) is the total amount of surface area of the periodontal pocket. PISA and PESA were calculated using a prepared spreadsheet (freely available from www.parsprototo.info), as previously described by Nesse et al. [13]. Oral bacterial measurements were determined with a device that provides quantification based on electrical impedance (Panasonic Healthcare Holdings Co., Ltd., Tokyo), according to previous reports [23, 24]. Samples were collected from the middle of the tongue surface using an exclusive applicator by a clinically experienced dental hygienist trained to minimize measurement errors. Oral bacterial counts were automatically determined by the device. Degree of xerostomia was determined using a Mucus oral moisture meter (Life Co., Ltd.) [25], with xerostomia defined based on a value  > 27, according to the manual provided by the manufacturer. Participants were queried regarding the frequency of dental visits, then categorized as “visited within one year” and “no visits for at least one year”.

### Statistical analysis

All analyses were performed using the statistical software package JMP 12.2 (SAS Institute Inc., Cary, NC, USA). Values are expressed as average, median, and standard error (SE) when an associated variable was a continuous variable, or as frequency and percentage clinically when that was a discrete variable. Univariate analysis was performed to evaluate between-group differences in regard to baseline characteristics, risk factors, oral assessment measures, and frequency of dental visits for both the HNC and other cancer groups. Comparisons between groups were made using Student’s T-test or a Mann–Whitney U test to compare two unpaired groups, and Fisher's exact probability test or a chi-squared test.

Multivariate logistic regression analysis was used to assess independent effects on HNC. Variables with a *P* value  < 0.05 in univariate analysis were subjected to multivariate analysis using a forced entry method. A Hosmer–Lemeshow goodness-of-fit test was used to evaluate the fit of the model. Propensity score-matched analysis was performed to eliminate the effects of clinical confounding factors, which were calculated using logistic regression analysis of 10 clinical parameters (i.e., age, gender, smoking status, alcohol consumption, hypertension, diabetes, hyperlipidemia, stroke, heart disease, previous episode of malignant tumor). A caliper width of 0.25 for standard deviations of propensity score was used for analysis. Univariate analysis was performed to evaluate risk factors that affected high PISA values in the HNC group, which was divided into high and low PISA subgroups based on the median value of 213. Finally, covariates with a *P* value  < 0.05 in univariate analysis and history of DM related to PISA were subjected to multivariate analysis with a forced-entry method. All analyses were two-sided, with *P* values  < 0.05 considered to be statistically significant.

## Results

Clinical characteristics of the 1030 patients retrospectively examined in this study are presented in Table 1. The mean age of the enrolled subjects was 64.3 ± 13.9 years. As for type of cancer, gastrointestinal cancer was the most common and noted in 274 cases, followed by hematological cancer in 180 and breast cancer in 150, while there were 142 cases of HNC. Similar to findings previously reported [26, 27], the HNC group had a significantly higher percentage of males, as well as patients with a history of smoking and drinking as compared to the others (*P* < 0.0001).

**Table 1:** Baseline clinical characteristics of cancer patients in this study (*n* = 1030)

Clinical parameter	
Age, years	64.3 ± 13.9
Gender, male/female	619/411
Smoking status, n (%)	
No	396 (38.4)
Former	433 (42.0)
Current	193 (18.7)
Alcohol consumption, n (%)	
No	428 (41.6)
Occasionally	256 (24.9)
Daily	335 (32.5)
Hypertension, n (%)	263 (25.5)
Diabetes mellitus, n (%)	139 (13.5)
Hyperlipidemia, n (%)	108 (10.5)
Stroke, n (%)	47 (4.6)
Heart disease, n (%)	68 (6.6)
Previous episode of malignant tumor	190 (18.5)
Cancer type, n (%)	
Gastroenterological cancer	274 (26.6)
Hematological cancer	180 (17.5)
Breast cancer	150 (14.6)
Head and neck cancer	142 (13.8)
Pulmonary cancer	131 (12.7)
Urology cancer	62 (6.0)
Gynecologic cancer	41 (4.0)
Brain cancer	26 (2.5)
Skin and musculoskeletal cancer	20 (1.9)
Unknown primary origin	4 (0.4)
Frequency of dental visits	
≥ 1 time/year	507 (49.2)
< 1 time/year	523 (50.8)

Following classification of the patients into the HNC (*n* = 142) and other cancer (*n* = 888) groups (Fig. 1), their clinical characteristics were compared. Results of univariate analysis of baseline characteristics to determine their association with HNC revealed gender, and smoking and drinking history to have significantly greater levels than other types of cancer (*P* < 0.0001) (Table 2).

**Table 2:** Comparison of clinical characteristics between HNC and other cancer groups

Parameter	HNC (*n* = 142)	Other (*n* = 888)	*P* value
Age, years	64.7 ± 10.8	64.3 ± 14.4	0.71
Gender, male/female	123/19	496/392	< 0.0001*
Smoking status, n (%)			< 0.0001*
No	30 (21.1)	367 (41.3)	
Former	73 (51.4)	362 (40.8)	
Current	39 (27.5)	154 (17.3)	
Alcohol consumption, n (%)			< 0.0001*
No	36 (25.4)	392 (44.1)	
Occasionally	29 (20.4)	228 (25.7)	
Daily	77 (54.2)	260 (29.3)	
Hypertension, n (%)	45 (31.7)	218 (24.6)	0.07
Diabetes mellitus, n (%)	22 (15.5)	117 (13.2)	0.46
Hyperlipidemia, n (%)	15 (10.6)	93 (10.5)	0.98
Stroke, n (%)	11 ( 7.7)	36 (4.1)	0.06
Heart disease, n (%)	6 ( 4.2)	62 (7.0)	0.20
Previous episode of malignant tumor	30 (21.1)	160 (18.0)	0.38

^*^Significant

Univariate analysis was also performed to compare clinical characteristics and oral environmental factors between the groups. Number of missing teeth, PISA, oral bacteria count, and xerostomia were significantly higher in the HNC group (*P* < 0.05, *P* < 0.001, *P* < 0.01, respectively), while presence of untreated dental caries, PESA, and frequency of dental visits was not significantly different between the groups (Table 3).

**Table 3:** Comparison of oral environmental factors between HNC and in other cancer groups

Parameter	HNC (*n* = 142)	Other (*n* = 888)	*P* value
Number of missing teeth ≥ 6 (%)	94 (66.2)	499 (56.2)	< 0.05*
Presence of untreated dental caries (%)	59 (41.6)	325 (36.6)	0.26
PESA (mm^2^), median (IQR)	1101.0 (804.2–1328.8)	1068.1 (794.0–1283.3)	0.35
PISA (mm^2^), median (IQR)	212.6 (113.9–319.0)	186.1 (65.0–271.1)	< 0.05*
Oral bacteria count (× 10^4^ CFU/mL)	1199.4 ± 1236.1	839.6 ± 1019.7	< 0.001*
Xerostomia	24.5 ± 5.1	25.6 ± 2.8	< 0.01*
Frequency of dental visits ≥ 1 time/year	63 (44.4)	444 (50.0)	0.25

^*^Significant

Multivariate logistic regression analysis using the forced entry method was performed for variables with *P* values  < 0.05 shown in univariate analysis (Table 4). The Hosmer–Lemeshow test for the model had a P value of 0.66, indicating a good fit. Those results showed number of missing teeth (odds ratio 1.72, 95% CI 1.15–2.56, *P* < 0.01), PISA (odds ratio 1.06, 95% CI 1.03–1.06, *P* < 0.05), and bacterial count (odds ratio 1.02, 95% CI 1.01–1.03, *P* < 0.01) to be independent factors related to HNC (Table 4). Next, to reduce selection bias, propensity score-matching was used to identify 139 matched pairs in the HNC and other cancer groups. Propensity score matching between the HNC and other cancer groups was performed using propensity scores generated from the 10 clinical factors. A total of 278 patients (139 matched pairs) with propensity score matching were evaluated using univariate analysis. None of the 10 clinical variables differed significantly between the groups. Among oral environmental factors, PISA and the number of oral bacteria were significantly higher in the HNC as compared to the other cancer group (*P* < 0.05) (Table 5).

**Table 4:** Multivariate logistic regression analysis to identify risk factors associated with HNC

Risk factor	Odds ratio	95% CI	*P* value
Number of missing teeth ≥ 6	1.72	1.15–2.56	< 0.01*
PISA	1.06	1.03–1.06	< 0.05*
Oral bacterial count	1.02	1.01–1.03	< 0.01*
Xerostomia	1.03	0.98–1.08	0.27

^*^Based on four factors included in analysis showing a *P* value < 0.05 by univariate analysis

^*^P < 0.05 (statistically significant in multivariate logistic regression using forced-entry method)

**Table 5:** Comparison of clinical characteristics between HNC and other cancer groups after propensity score-matching

Parameter	HNC (*n* = 139)	Other (*n* = 139)	*P* value
Age, years	64.7 ± 12.4	65.5 ± 13.5	0.57
Gender, male/female	120/19	120/19	1.00
Smoking status, n (%)			0.62
No	29 (20.9)	30 (21.6)	
Former	71 (51.1)	77 (55.4)	
Current	39 (28.0)	32 (23.0)	
Alcohol consumption, n (%)			0.72
No	36 (25.9)	36 (25.9)	
Occasionally	28 (21.1)	23 (16.6)	
Daily	75 (54.0)	80 (57.5)	
Hypertension, n (%)	45 (32.4)	51 (36.7)	0.45
Diabetes mellitus, n (%)	22 (15.8)	25 (18.0)	0.63
Hyperlipidemia, n (%)	15 (10.8)	19 (13.7)	0.46
Stroke, n (%)	11 (7.9)	7 (5.0)	0.33
Heart disease, n (%)	6 (4.3)	2 (1.4)	0.14
Previous episode of malignant tumor, n (%)	29 (20.9)	26 (18.7)	0.65
Number of missing teeth ≥ 6 (%)	89 (54.3)	75 (45.7)	0.11
Presence of untreated dental caries, n (%)	58 (49.2)	60 (50.9)	0.80
PESA (mm^2^), median (IQR)	1108.6 (822.1–1350.5)	1108.1 (776.5–1298.3)	0.40
PISA (mm^2^), median (IQR)	216.4 (115.3–322.4)	160.9 (55.1–301.8)	< 0.05*
Oral bacteria count (× 10^4^ CFU/mL)	1188.1 ± 1222.3	881.1 ± 1199.7	< 0.05*
Xerostomia	24.6 ± 5.4	25.1 ± 2.4	0.06
Frequency of dental visits ≥ 1 time/year, n (%)	48 (52.2)	59 (51.3)	0.90

^*^Significant

PISA provides quantification of the present inflammatory burden posed by periodontal disease in individual subjects [13, 20]. To evaluate risk factors for a high PISA value in association with HNC, those patients were divided into the high (*n* = 71) and low (*n* = 71) PISA subgroups according to the median value of 213, then clinical characteristics and oral environmental factors were compared. Values for PESA were excluded, as those were explanatory variables not independent of PISA. Results of univariate analysis of risk factors with effects on a high PISA value in the HNC patients are presented in Table 6. Age, smoking status, and presence of untreated dental caries were significantly more frequent in the high as compared to low PISA subgroup (*P* < 0.005, *P* < 0.005, *P* < 0.0001, respectively). On the other hand, there were no significant differences in regard to gender, alcohol consumption, hypertension, diabetes, hyperlipidemia, stroke, heart disease, history of malignancy, missing teeth, oral bacterial count, xerostomia, or frequency of dental visits. The three explanatory variables with *P* values  < 0.05 in univariate analysis and also diabetes mellitus, as it has been reported to have a relationship with PISA [28], were then entered into multivariate logistic regression analysis using a forced entry method (Table 7). Those results showed current smoker (odds ratio 7.51, 95% CI 1.63–34.71, *p* = 0.0098) and presence of untreated caries (odds ratio 3.33, 95% CI 1.23–9.00, *p* = 0.0179) to be independent risk factors for high PISA in the present HNC patients.

**Table 6:** Comparison of clinical characteristics between low and high PISA subgroups of HNC patients

Parameter	PISA < 213 (*n* = 71)	PISA ≥ 213 (*n* = 71)	*P* value
Age, years	67.6 ± 9.9	61.8 ± 10.9	< 0.005*
Gender, male/female	60/11	63/8	0.45
Smoking status, n (%)			< 0.005*
No	18 (25.4)	11 (15.5)	
Former	41 (57.7)	30 (42.3)	
Current	10 (14.1)	29 (40.8)	
Alcohol consumption, n (%)			0.99
No	18 (25.4)	18 (25.4)	
Occasionally	14 (19.7)	14 (19.7)	
Daily	37 (52.1)	38 (53.5)	
Hypertension, n (%)	25 (35.2)	20 (28.2)	0.37
Diabetes mellitus, n (%)	10 (14.1)	12 (16.9)	0.64
Hyperlipidemia, n (%)	10 (14.1)	5 (7.0)	0.17
Stroke, n (%)	6 (8.5)	5 (7.0)	0.75
Heart disease n (%)	3 (4.2)	3 (4.2)	1.00
Previous episode of malignant tumor	17 (23.9)	13 (18.3)	0.41
Number of missing teeth ≥ 6 (%)	56 (78.8)	38 (53.5)	0.47
Presence of untreated dental caries (%)	18 (25.4)	41 (58.0)	< 0.0001*
Oral bacteria count (× 10^4^ CFU/mL)	1036.6 ± 1101.1	1347.5 ± 1337.8	0.14
Xerostomia	25.2 ± 2.9	26.0 ± 2.7	0.09
Frequency of dental visits ≥ 1 time/year	33 (46.3)	30 (41.8)	0.64

^*^Significant

**Table 7:** Multivariate logistic regression analysis to identify independent risk factors affecting high PISA value in HNC patients

Risk factor	Odds ratio	95% CI	*P* value
Age	0.64	0.05–7.63	0.73
Smoking status			
Former	1.03	0.31–3.40	0.96
Current	7.51	1.63–34.71	< 0.01*
Diabetes mellitus	1.08	0.28–4.12	0.90
Presence of untreated dental caries	3.33	1.23–9.00	< 0.05*

Analysis based on five factors showing a *P* value < 0.05 was performed using a univariate Mann–Whitney U test, a chi-squared test, or univariate logistic regression, and one factor considered to be clinically relevant

^*^P < 0.05 (statistically significant in multivariate logistic regression via forced-entry method)

## Discussion

For the present study, the assessment index PISA was utilized to compare periodontal inflammation between HNC and other types of cancer, which showed that PISA was significantly associated with HNC as compared to other types of cancer. PISA uses the sum of probing pocket depth of BOP-positive areas to determine present inflammatory burden, as gingival bleeding is an important sign of inflammation and supports identification of actively inflamed periodontal tissues, thus it can be utilized to distinguish between actively inflamed and non-inflamed or healed dentition [13, 20]. A recent report showed that PISA was associated with CRP level in serum samples obtained from septuagenarian subjects. CRP indicates systemic inflammation [29] and patients with severe periodontitis have been reported to have an increased level in serum [15, 16]. Therefore, PISA is considered to be a useful marker for estimating the effect of periodontitis on the whole body and considered to be more sensitive than severity detected in conventional periodontal examinations [19]. Oral epithelium disruption caused by development of periodontitis results in release of inflammatory cytokines, such as TNF and IL-1 β [30]. Those cytokines are generally detected in the first phase of carcinogenesis, and their overexpression promotes tumor formation and development of cancer [31, 32]. Periodontitis development can induce excessive production of reactive oxygen species from inflammatory cells, a type of oxidative stress known to be associated with DNA damage [8, 31]. Additionally, recent studies have shown a direct role of periodontal bacteria such as *P. gingivalis* in carcinogenesis [8, 33]. On the other hand, PESA was not significantly different between the HNC and other cancer groups. PESA accurately quantifies the surface area of pocket epithelium when the gingival margin is located at or below the cement enamel junction, and some parts that comprise PESA consist of healthy epithelium that does not contribute to inflammatory burden [14]. The results obtained in the present study indicate that HNC patients are presented with greater levels of gingival inflammation in periodontal tissues as compared with patients affected by other types of cancer, which may be associated with development and progression of HNC.

Univariate analysis results showed that PISA, number of missing teeth, oral bacterial count, and xerostomia were significantly associated with HNC as compared with other types of cancer in the present patients, while multivariate logistic regression analysis indicated that number of missing teeth, PISA, and bacterial count were independent factors related to HNC. In several previous cohort and case control studies, tooth loss was used as a surrogate parameter for periodontal disease and found to be a significant dependent risk factor for HNC [18]. Although number of missing teeth does not necessarily indicate the presence of periodontitis, it has been shown to be related to dental visits and a history of poor oral hygiene [34, 35]. On the other hand, oral bacterial count has been found to be dependent on oral hygiene condition such as xerostomia and have an influence on perioperative complications during cancer treatment [36]. Also, oral hygiene management during cancer treatment or palliative therapy has been reported to result in a decreased oral bacterial count and improve complications related to xerostomia [37, 38]. The present results indicate that HNC patients have worse oral hygiene as compared to patients with other types of cancer, thus prompt oral assessment and planning of oral hygiene management is needed at the time of HNC diagnosis.

Previous studies have shown that HNC is associated with periodontal diseases [9–12], which constantly contribute to systemic inflammation, resulting in increased plasma levels of proinflammatory cytokines, acute phase proteins, and other proteinases. Those inflammatory processes can generate free radicals and active intermediates, which causes oxidative stress related to DNA damage [8, 9]. Furthermore, periodontopathic bacteria have been reported to have a direct role in carcinogenesis development [8]. On the other hand, patients with cancer often have decreased levels of activities of daily living (ADL) and instrumental activities of daily living (IADL) [39]. Therefore, brushing deficiency due to a decrease in IADL related to onset of HNC may secondarily promote poor oral hygiene, thus leading to periodontal inflammation indicated by PISA. To elucidate the mechanism of the relationship of HNC with periodontal inflammation, oral environment factors such as oral hygiene condition and periodontal inflammation prior to the onset of HNC must be examined.

Current smoking status was found to be an independent risk factor related to a high PISA value in the present HNC patients. Smoking is known to be the most significant risk factor for HNC development [40, 41]. Additionally, smoking is known to affect mechanisms related to the pathogenesis and development of periodontal diseases, including decreasing gingival perfusion, which restricts nutrients and oxygen delivery, thus suppressing immune response, and inhibiting morphological and functional recovery of the periodontium, and increasing the infectivity of oral microbiota. These factors combine to promote disruption of wound healing and accelerate development of periodontal disease [42]. Some clinical and epidemiological studies have also shown that smokers have fewer gingival bleeding pockets, as well as a suppressive effect of smoking on gingival bleeding [43–45]. PISA indicates the surface area of bleeding pocket epithelium in square millimeters [13], thus the present results appear to contradict previous reports regarding the relationship of gingival bleeding and smoking. However, Park et al. demonstrated that the quantity of current smoking was related to increased PISA value in a dose-dependent manner [14]. The present findings indicating that a high PISA value is affected by current smoking in HNC patients may imply a relationship with development of HNC and periodontitis.

Untreated caries was also found to be an independent risk factor related to a high PISA value in the HNC group. Indeed, some cross-sectional studies have shown a positive association between prevalence of untreated dental caries and severity of periodontal disease [46, 47], with the latter report noting that caries and periodontal disease should generally be considered as related risk factors during oral hygiene maintenance conducted during a regular dental visit. Furthermore, untreated caries can increase plaque retention and predispose the patient to periodontal disease [48]. Oral hygiene management has been reported to prevent various complications, such as oral mucositis and infection, during treatment for HNC [49–51]. Thus, improvement of poor oral health by caries and periodontal treatments in HNC patients prior to undergoing cancer therapy can decrease gingival inflammation, and that may reduce complications related to cancer treatment.

A previous report noted that low socioeconomic status (SES) was a risk factor strongly associated with HCN, with current smoking more strongly associated with HCN among households with low income [52]. Furthermore, low SES has been shown to be related to dental caries, periodontal diseases, and poor oral hygiene [53–55]. Although the SES of the present HCN patients was not examined, low SES may be a risk factor for increased PISA in HNC patients.

The present investigation has several limitations. First, PISA shows the current periodontal inflammatory burden caused by periodontal disease but does not indicate inflammation occurring at the onset of head and neck cancer. While there are no known markers that show continuous periodontal inflammation from the onset of HNC, the large numbers of missing teeth and high PISA values noted in HNC patients suggest repeated occurrence of a high level of gingival inflammation. Second, a comparison of periodontal inflammation markers such as PISA between HNC patients and healthy subjects was not performed. On the other hand, comparisons of markers related to periodontitis between patients with various types of cancer and healthy subjects have been examined in previous studies, which showed that periodontitis is a risk factor related to cancer occurrence including HNC [1–4, 9–12]. Therefore, poor oral health and a high level of gingival inflammation may have a greater association with development and progression of HNC as compared to other cancer types. Third, PISA is a new periodontal index and still has a low degree of recognition. However, several investigators have recently shown associations with systemic disease, physical status, and onset of complication after cancer treatment [19, 21, 22, 56–60]. In the future, PISA may become a representative marker for examining the relationship between periodontitis and systemic diseases. Fourth, the present analysis has intrinsic limitations related to its retrospective nature, particularly regarding data completeness. Notably, even though human papillomavirus (HPV) infection is a risk factor for HNC, detection of HPV16 DNA or E6/E7mRNA expression in oropharyngeal swab samples from affected patients was not performed [61, 62]. Fifth, this was a single-center study, which limits generalization of the findings. Sixth, the present results are limited by use of multiple comparisons. Factors that showed a *P* value  < 0.05 in univariate analysis and clinically relevant factors were subjected to multivariate logistic regression analysis. Causal factors can be shown in such analysis results by chance when several variables are examined together. Finally, it has been reported that oral bacterial count determined with the device used in the present study is associated with complications occurring after surgery for cardiovascular disease and during chemotherapy for breast cancer [24, 36]. However, differences regarding bacterial species on the tongue surface between patients with HNC and other types of cancer were not clarified in the present study. An investigation performed to identify specific bacteria related to HNC is needed.

In conclusion, this is the first known study to demonstrate that PISA, number of missing teeth, and oral bacteria count were significantly associated with HNC as compared with other types of cancer. Furthermore, smoking and untreated caries were shown to be independent risk factors affecting a high PISA value in HNC cases. Patients with HNC are often presented with a high level of periodontal inflammation and poor oral hygiene as compared with those with other cancer types. Therefore, prompt oral assessment and planning of oral hygiene management is necessary at the time of HNC diagnosis.

### Supplementary Information

**Additional file 1:** **Supplemental Data 1.** Comparisons of clinical characteristics between oral/oropharyngeal and other cancer type groups including HNC without oral/oropharyngeal cancer. **Supplemental Data 2.** Comparisons of oral environmental factors between oral/oropharyngeal and other cancer type groups including HNC without oral/oropharyngeal cancer. **Supplemental Data 3.** Multivariate logistic regression analysis to identify risk factors associated with oral/oropharyngeal cancer. **Supplemental Data 4.** Comparisons of clinical characteristics and oral environmental factors between oral/oropharyngeal cancer group and HNC without oral/oropharyngeal cancer group.

## Data Availability

Raw data obtained in this study are not publicly available due to ethical restrictions, though are available for any author who wishes to collaborate with the present authors.
