# Supplementary material for: Head and neck cancer patients show poor oral health as compared to those with other types of cancer
Source: BMC Oral Health. 2023 Sep 6;23:647. doi: 10.1186/s12903-023-03356-6 (PMC10483752; doi:10.1186/s12903-023-03356-6)
Supplement: Supplementary file 1 — Additional file 1: Supplemental Data 1. Comparisons of clinical characteristics between oral/oropharyngeal and other cancer type groups including HNC without oral/oropharyngeal cancer. Supplemental Data 2. Comparisons of oral environmental factors between oral/oropharyngeal and other cancer type groups including HNC without oral/oropharyngeal cancer. Supplemental Data 3. Multivariate logistic regression analysis to identify risk factors associated with oral/oropharyngeal cancer. Supplemental Data 4. Comparisons of clinical characteristics and oral environmental factors between oral/oropharyngeal cancer group and HNC without oral/oropharyngeal cancer group. [file 12903_2023_3356_MOESM1_ESM.docx]

Supplemental Data 1 (for reviewer).

Comparisons of clinical characteristics between oral/oropharyngeal and other cancer type groups including HNC without oral/oropharyngeal cancer.

| Parameter | Oral/oropharyngeal  (n=79) | Others  (n=951) | P value |
| --- | --- | --- | --- |
| Age, years | 63.0±10.1 | 64.4±14.2 | 0.39 |
| Gender, male/female | 68/11 | 551/400 | <0.0001* |
| Smoking status, n (%) |  |  | <0.03* |
| No | 18 (23.4) | 394 (41.4) |  |
| Former | 40 (52.0) | 382 (40.2) |  |
| Current | 19 (24.7) | 170 (17.9) |  |
| Alcohol consumption, n (%) |  |  | <0.03* |
| No | 21 (25.4) | 407 (42.8) |  |
| Occasionally | 2 (20.4) | 237 (24.9) |  |
| Daily | 36 (54.2) | 301 (31.6) |  |
| Hypertension, n (%) | 20 (25.3) | 243 (25.6) | 0.96 |
| Diabetes mellitus, n (%) | 10 (12.7) | 129 (13.6) | 0.81 |
| Hyperlipidemia, n (%) | 6 (7.6) | 102 (10.7) | 0.36 |
| Stroke, n (%) | 6 (7.6) | 41 (4.3) | 0.21 |
| Heart disease, n (%) | 5 (6.3) | 63 (6.6) | 0.93 |
| Previous episode of malignant tumor | 16 (20.3) | 174 (18.3) | 0.67 |

*Significant

Supplemental Data 2 (for reviewer).

Comparisons of oral environmental factors between oral/oropharyngeal and other cancer type groups including HNC without oral/oropharyngeal cancer

| Parameter | Oral/oropharyngeal cancer  (n=79) | Other cancers  (n=951) | P value |
| --- | --- | --- | --- |
| Number of missing teeth ≥6 (%)  Presence of untreated dental caries (%)  PESA (mm^2^), median (IQR)  PISA (mm^2^), median (IQR)  Oral bacteria count (x10^4^ CFU/mL)  Xerostomia  Frequency of dental visits ≥1 time/year | 53 (67.1)  32 (40.5)  1101.6 (831.8-1367.7)  208.2 (123.7-288.4)  1324.4±1300.7  25.5±3.0  31 (39.2) | 540 (56.8)  352 (37.0)  1071.7 (795.6-1285.9)  203.6 (73.4-302.6)  851.8±1026.9  24.6±5.0  476 (50.1) | <0.05*  0.47  0.25  <0.05*  <0.001*  0.11  0.87 |

*Significant

Supplemental Data 3 (for reviewer).

Multivariate logistic regression analysis to identify risk factors associated with oral/oropharyngeal cancer.

| Risk factor | Odds ratio | 95% CI | P value |
| --- | --- | --- | --- |
| Number of missing teeth ≥6  PISA  Oral bacteria count | 1.78  1.01  1.02 | 1.20-2.65  1.00-1.01  1.00-1.03 | <0.01*  <0.05*  <0.01* |

Based on three factors included in analysis showing a P value <0.05 by univariate analysis. *P <0.05 (statistically significant in multivariate logistic regression using forced-entry method).

Supplemental Data 4 (for reviewer).

Comparisons of clinical characteristics and oral environmental factors between oral/oropharyngeal cancer group and HNC without oral/oropharyngeal cancer group

| Parameter | Oral/oropharyngeal cancer  (n=79) | HNC without oral/oropharyngeal cancer  (n=63) | P value |
| --- | --- | --- | --- |
| Age, years | 63.0±10.1 | 66.0±10.9 | 0.10 |
| Gender, male/female | 68/11 | 52/8 | 0.92 |
| Smoking status, n (%) |  |  | 0.73 |
| No | 18 (23.4) | 11 (18.3) |  |
| Former | 40 (52.0) | 31 (51.7) |  |
| Current | 19 (48.7) | 20 (33.9) |  |
| Alcohol consumption, n (%) |  |  | 0.22 |
| No | 21 (27.3) | 15 (25.0) |  |
| Occasionally | 20 (26.0) | 8 (13.6) |  |
| Daily | 38 (48.1) | 37 (62.7) |  |
| Hypertension, n (%) | 22 (27.8) | 23 (38.3) | 0.17 |
| Diabetes mellitus, n (%) | 10 (12.7) | 12 (20.0) | 0.24 |
| Hyperlipidemia, n (%) | 7 (8.9) | 8 (13.3) | 0.37 |
| Stroke, n (%) | 6 (7.6) | 5 (8.3) | 0.87 |
| Heart disease, n (%) | 5 (6.3) | 1 (1.7) | 0.18 |
| Previous episode of malignant tumor, n (%) | 16 (20.3) | 13 (21.7) | 0.84 |
| Number of missing teeth ≥6 (%) | 50 (65.8) | 39 (66.1) | 0.97 |
| Presence of untreated dental caries, n (%) | 33 (41.8) | 25 (41.7) | 0.99 |
| PESA (mm^2^), median (IQR) | 1076.1 (978.6-1173.5) | 1045.6 (875.4-1215.7) | 0.74 |
| PISA (mm^2^), median (IQR) | 276.2 (209.4-343.0) | 314.5 (231.7-397.5) | 0.47 |
| Oral bacteria count (x10^4^ CFU/mL) | 1324.4±1300.7 | 947.3±1019.7 | 0.08 |
| Xerostomia | 25.5±3.0 | 25.7±2.7 | 0.68 |
| Frequency of dental visits ≥1 time/year, n (%) | 31 (39.2) | 17 (28.3) | 0.17 |
